# Quantitative Susceptibility Mapping: Translating an Investigative Research Tool into High Volume Clinical Diagnostic Imaging

**DOI:** 10.3390/diagnostics12122962

**Published:** 2022-11-26

**Authors:** Matthew T. Borzage, Eamon K. Doyle, Chia-Shang J. Liu, Marvin D. Nelson, Stefan Blüml, John C. Wood, Benita Tamrazi

**Affiliations:** 1 Department of Pediatrics, Children’s Hospital of Los Angeles, Keck School of Medicine, University of Southern California, Los Angeles, CA 90027, USA; 2 Department of Radiology, Children’s Hospital of Los Angeles, Keck School of Medicine, University of Southern California, Los Angeles, CA 90027, USA; 3 Department of Biomedical Engineering, Viterbi School of Engineering, University of Southern California, Los Angeles, CA 90027, USA; 4 Division of Cardiology, Department of Pediatrics and Radiology, Children’s Hospital of Los Angeles, Los Angeles, CA 90027, USA

**Keywords:** QSM, iron, MRI, processing, translation

## Abstract

Quantitative susceptibility mapping (QSM) is an MRI-based technique for iron quantification of targeted tissue. QSM provides information relevant to clinicians in a broad range of diagnostic contexts, including sickle cell disease, inflammatory/demyelinating processes, and neoplasms. However, major MRI vendors do not offer QSM post-processing in a form ready for general use. This work describes a vendor-agnostic approach for scaling QSM analysis from a research technique to a routine diagnostic test. We provide the details needed to seamlessly integrate hardware, software, and clinical systems to provide QSM processing for a busy clinical radiology workflow. This approach can be generalized to other advanced MRI acquisitions and analyses with proven diagnostic utility, yet without crucial MR vendor support.

## 1. Introduction

Quantitative susceptibility mapping (QSM) is an attractive but underutilized MRI diagnostic modality that quantifies magnetic susceptibility. Susceptibility is related to tissue iron content and extrinsic contrast mechanisms. QSM acquisitions collect gradient recalled echoes (GRE) at multiple echo times and use the phase evolution to quantify the local magnetic field [1]. The magnitude images from QSM acquisitions yield anatomic images suitable for routine clinical brain MRI. Post-processing the magnitude and phase images from QSM acquisitions can yield susceptibility weighted images in addition to the susceptibility maps. Previous studies have demonstrated the quantitative nature of QSM in phantoms [2] and in vivo studies [3]. Thus, QSM analysis is a “free-lunch” from an image acquisition time perspective, thereby making it highly desirable in busy clinical environments. Furthermore, the QSM acquisition does not require special MRI vendor hardware or software, thereby making it broadly applicable to existing MRI systems. QSM imaging does not require any contrast injections, which is important given the recent data on gadolinium retention in the brain. The QSM images can be used either qualitatively in everyday clinical practice, or quantitatively if the susceptibility of specific regions is relevant to a patient.

However, QSM requires a technical analysis pipeline and infrastructure for post-processing that converts the raw images into susceptibility maps. This processing takes place after the images are acquired and involves solving an ill-posed three-dimensional dipole inversion. MR vendors ideally would incorporate QSM processing into their commercial products, however, at the current time; there is no such option available. To our knowledge, Canon and Siemens each have a “work-in-progress” QSM processing solution and Philips plans to release a clinical science patch, but none of these is available for clinical use. Without their support, the QSM analysis pipeline has been too cumbersome to incorporate into a fast-paced radiology practice.

Further, a variety of approaches to QSM with different sequence parameters and reconstructions have been attempted. The UK Biobank demonstrated QSM image processing in a dataset of 35,273 participants studying brain structure, but the data was not used in a clinical setting and only utilized two echo times as the protocol [4]. Other studies have reconstructed QSM images with different numbers of echo times [5] and non-cartesian readouts [6]. QSM has been integrated into certain clinical scenarios, but the process of performing the automatic image reconstruction and pipeline integration was not described [7]. Because numerous approaches to reconstructing QSM images exist and each come with their own advantages and disadvantages [8,9,10,11,12,13], the ability to develop and test new protocols and reconstruction techniques beyond what is provided by vendors is critical to advance the field of QSM.

New imaging contrasts, often achieved using offline reconstruction tools, are a challenge to deploy in the clinical setting. For example, at our institution, the offline reconstruction process is at odds with the need to finalize imaging reports within a certain time following image acquisition for billing purposes. Integration with clinical systems is administratively and technically challenging. Thus, despite widespread interest, clear clinical potential, and a simple acquisition, the complicated steps for processing QSM imaging exclude it from the clinical standard of care imaging. We aim to close this significant gap between the current research and clinical imaging worlds.

### Clinical Utility of Quantitative Susceptibility Mapping

Iron is an essential element for normal metabolism, but excess iron is potentially toxic. Disrupted brain iron homeostasis causes deleterious effects [14]. Iron deficiency anemia impairs cognitive and emotional development [15]. Decreased brain iron is associated with autism [16]. In contrast, increased brain iron is observed in Parkinson’s Disease [17,18], Amyotrophic Lateral Sclerosis [19], Alzheimer’s Disease [20], and sickle cell disease [21].

Iron’s proinflammatory effects in the central nervous system has generated great interest in the clinical utility of QSM for assessing neurodegeneration and aging [22] as well as assessing treatment efficacy and prognostication [23]. QSM accurately assays brain iron [5] and facilitates the diagnosis of iron related neurodegeneration. Either acute or chronic inflammation increase iron deposition in the brain and accelerate neurodegeneration [24]. In the setting of Parkinson’s Disease, QSM imaging quantifies iron deposition associated with disease burden and assesses the response to chelation therapies that might halt the progression of symptoms [25]. QSM imaging is poised to dominate the clinical imaging world as the method of choice to identify, quantify, and follow longitudinally the changes that occur over time for many patients with either normal aging or pathological accelerated neurodegeneration.

**Figure 1:**
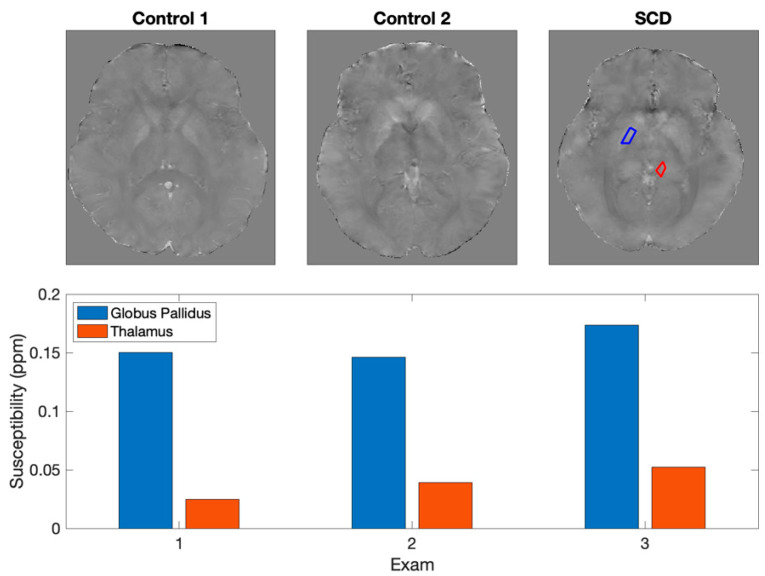
Representative QSM maps from two healthy controls compared to one subject with sickle cell disease demonstrating elevated iron accumulation. This cross-sectional data shows the utility of imaging many patients, which provides a reference between patients with healthy brains and matched patients with elevated iron accumulation.

**Figure 2:**
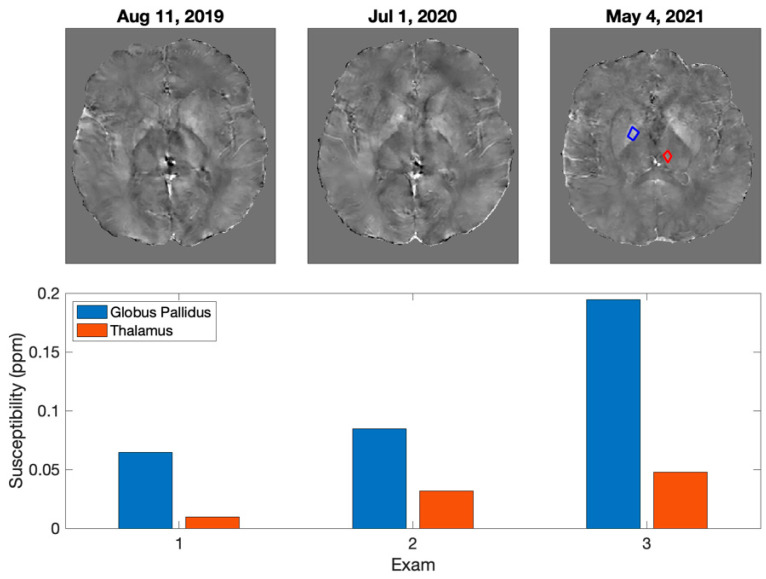
Example of one patient over time demonstrating increased iron accumulation following cranial radiation therapy. The longitudinal data over 632 days shows the utility of maintaining sequential routine QSM imaging and the ability to detect increases in iron over time. The patient (13 years old at diagnosis in 2019) underwent cranial radiation therapy involving whole ventricular zone coverage at 3600 cGy and a pineal boost at 1800 cGy concluding on 8/2019. Subsequent serial imaging demonstrates pathological increased accumulation of iron in the globus pallidus in an accelerated timeline as would be expected for this age.

Specific pathologies in the pediatric population lead to accelerated brain iron deposition. For example, patients with Sickle Cell Disease (Figure 1) develop greater iron accumulation in deep brain nuclei [21]. Other children may have iatrogenically increased iron deposition. For example, children with brain tumors who receive cranial radiation therapy (Figure 2) might develop greater brain iron accumulation yielding neurocognitive morbidities [26,27,28]. Future prospective studies should evaluate associations between their disease, treatment, brain iron deposition, and long-term neurocognitive deficits. 

Thus, QSM images are readily acquired, add no time to the patient scan, and demonstrate strong clinical potential but vendors have not adopted and promulgated QSM images. Institutions must therefore remove the remaining barriers that prevent adoption of QSM. This complex process may be time-consuming and require a hierarchy of stakeholder approval in Radiology and Information Services. 

Implementing new clinical imaging protocols generally requires agreement from the Department of Radiology. Key factors in the clinical approval process include changes to the MR exam duration, training technologists to acquire the images, addressing scheduling logistics if the sequences are only available on some scanners, the burden on radiologists in terms of time for interpretation and reporting. 

Information Services Nonstandard data processing and data flow within clinical data systems requires approval the Department of Information Services (IS). Key factors in the administrative approval process include potential disruption to clinical workflows, protecting hospital systems from internal and external security issues, provisioning of computing resources, risk aversion to non-commercial product implementations, and lack of enterprise support.

The objectives of this manuscript are to provide our experience overcoming these clinical and data transmission barriers. We provide our standard QSM acquisition and processing approaches, to facilitate others. We also provide examples of clinical and research imaging efforts improved by us deploying QSM imaging. We believe this work serves as a paradigm for how to bridge between imaging research and clinical diagnostic imaging, thereby extrapolating to other valuable yet-unsupported MR sequences.

## 2. Materials and Methods

QSM imaging requires four components: image acquisition, data management, image processing, and results analysis. Detailed reports on QSM acquisition and processing approaches are the focus of other reports [21]. We anticipate new scanner hardware or post processing approaches will improve upon those specific methods. In contrast, we anticipate approaches for data management and report analyses will remain much more stable.

### 2.1. Image Acquisition

The image acquisition is the most straightforward portion of the effort in the construction of a QSM pipeline. Our QSM sequence is a 3D multi-echo gradient echo (GRE) acquisition with flow compensation, 4 echoes with uniform TE spacing of 6.9 ms and a minimum TE of 5.7 ms. The QSM acquisition used at our institution has a total runtime of approximately 4.5 min to achieve whole brain coverage and can yield standard both GRE and susceptibility weighted images (SWI). At our institution, the SWI and GRE series are pushed to the clinical PACS prior to the completion of the QSM reconstruction and reviewed clinically even if a QSM reconstruction is ultimately not completed. Additional parameters are summarized in Table 1. Both magnitude and phase images must be reconstructed and saved for analysis. For comparison, research imaging protocols with GRE acquisitions include the ADNI longitudinal study of neurodegeneration (duration 4.2 min, cannot be processed into QSM) [29], and the well-known MarkVCID study of small vessel disease (5.2–6.3 min, can be processed into QSM) [30]. 

Key Considerations and Advice:

- We recommend deploying image acquisitions with a minimum acceptable level of quality as a first step. Deploying the QSM acquisition first allows a new user to verify their scanners provide the minimum image quality; new users can address concerns about scanner time, technologist training, and interpretability by radiologists; users can also use the resulting data to test their approaches for data management, image processing, and results analyses.
- If the scanner cannot provide the basic image quality, then the new user may need to work with their vendor to address their limitations. For example, GE does not provide access to the phase images without research mode that allows setting the “rhrcctrl” control variable.
- Calculate the imaging time dedicated to the combination of gradient echo and susceptibility weighted imaging in your current clinical protocols. A QSM sequence with minimal increase in scan duration with respect to that benchmark will facilitate sequence approval.
- Using a QSM sequence with an equal TR, voxel size and field of view with at least one echo time equal to the current standard-of-care GRE imaging at your institution will mitigate any contrast differences between the old (GRE) and new (QSM) data acquisitions.
- Using a QSM sequence with published parameters (e.g., MarkVCID, this manuscript, etc.) will facilitate intra-institution exchange of data.
- Changes to imaging parameters will make longitudinal analyses considerably more difficult.
- Have a plan for versioning your acquisitions and all parameters used for each acquisition.

### 2.2. QSM Data Management

The host computer of the MRI system saves QSM data at the time it is acquired. We strongly advise the user to move their data to a durable and redundant system such as the clinical PACS or a research PACS. Each institution may have one or several suitable data storage locations. Example scripts for data handling can be found in the supplementary code repository.

#### 2.2.1. Moving QSM Data 

A manual export to a USB drive by the user is often the simplest method to move QSM data to the data storage location. The process is convenient and requires little or no configuration on the MR host computer. However, the manual nature of the data export means that the processes of exporting data, uploading data, and naming files and folders all require human intervention. Thus, these manual processes are error prone, time consuming, and lead to data loss. Therefore, we do not recommend manual data exporting when handling large numbers of datasets. A better alternative is for the user to work with DICOM C-MOVE/C-GET protocols to move QSM data from the MRI system to the data storage location. The process requires more configuration of the computer systems, but will minimize the manual work, data loss, and the delay between acquisition and processing.

We move our QSM data from our MR host computer to the clinical PACS system first. This approach prevents data loss and eliminates variations in the clinical imaging workflow as experienced by our imaging staff. This approach is particularly valuable if the user is deploying QSM imaging on multiple scanners. Moving to clinical PACS first simplifies the process of moving QSM data to a single location. Moreover, radiologists can provide clinical interpretation of the GRE magnitude or susceptibility images within the QSM imaging datasets.

We move a second copy of our QSM data from clinical PACS to a research PACS system immediately after it arrives in the clinical PACS system. This approach minimizes the research interaction with the clinical PACS to a C-GET command to pull desired images. This approach also allows us to work with a PACS system suitable for automated data export and automated image processing and reconstruction. We selected Orthanc, an open-source DICOM-compliant server that also offers a Representational State Transfer (REST) Application Programming Interface (API) to facilitate integration with processing pipelines. Our Orthanc server is configured as a remote modality in the hospital clinical PACS, allowing datasets from the clinical PACS to be pushed to our system for processing. 

We also move a third copy of our QSM data from research PACS to an additional redundant network file system. The objective of this third copy is to ensure we have an archival copy of our QSM data in the unlikely event that both the clinical and research PACS data are damaged. 

#### 2.2.2. Storing QSM Imaging Data 

A single large hard drive by is often the simplest location to store QSM data. A hard drive storage solution is inexpensive, convenient, requires little or no configuration. and is compatible with the USB-based data moving solution for moving data. However, a single external hard drive represents a potential catastrophic failure point for the project. Data stored on a single hard drive is prone to total data loss and creates a risk for a large PHI-breach. Scaling the data management from a pilot project to a long-term work thread requires a research PACS system that is reliable, of sufficient size, and accessible to the appropriate team members, and robust to failure. Our QSM data is approximately 250 MB per scan but we recommend 1.25 GB per exam to save other MR sequences and QSM data at interim processing steps. 

We recommend at least using a dedicated network attached storage device that uses some type of RAID (redundant disk protection) so that you can remain online when a disk fails. It is preferable to consider a clustered file system such as Ceph that will allow horizontal scaling as the project grows and provides features such as encryption at rest. Using a clustered file system allows all project computation needs, including raw data, database, and virtual machine disk images, to live in the same logical space, allowing for a single point of backup and maintenance.

#### 2.2.3. Non-Imaging Data Storage and Management 

One or more spreadsheets are commonly used to store lists of key patients and information on their imaging and clinical histories. This solution is easy for nontechnical personnel to create and maintain. However, a spreadsheet lacks adaptability and auditability of relational databases. Our non-imaging data was initially maintained in spreadsheets designed for transfer to relational databases. Our non-imaging data was migrated to a relational database in MySQL with a phpMyAdmin interface. Other sources of important non-imaging data include informal emails and written to-do lists. We suggest adopting software development management tools exist and can be deployed to collect and track these efforts.

#### 2.2.4. Key Considerations and Advice

- Minimize and eliminate manual data handling practices early.
- If your clinical PACS can be involved in your workflow, then it will simplify the overall process. If you are unable to use your clinical PACS, then both moving and storing QSM data become more difficult.
- If exporting from scanners is a manual data process, then simplify this workflow by minimizing or eliminating choices on where the data can be sent.
- If multiple data destinations exist, have a plan to monitor them for accidental data transfers.
- Monitor for important non-imaging data and have a plan on how and when to systematize its collection and storage.

### 2.3. Image Processing

Image preprocessing comprises all of the scripts that identify and prepare data for processing, and image post processing comprise all scripts that push processed data to various destinations. These general-purpose scripts can be used to analyze many different types of image. In contrast, the QSM image processing are the idiosyncratic reconstruction and analysis scripts unique to our QSM data.

### 2.4. General Image Preprocessing

Our preprocessing code monitors the research PACS. Our preprocessing scripts identify data in our Research PACS that are suitable for analysis, when it finds them, it adds details on these images to our relational database. A second preprocessing script reviews the database for unprocessed images, when it finds them, it annotates the database entry to indicate the data has been retrieved for processing, downloads the images using the Orthanc REST API, and then sent to the QSM analysis scripts.

#### 2.4.1. QSM Image Processing

Our processing pipeline is based in MATLAB, and includes importing the QSM DICOM images, brain extraction (BET2), MEDI-based Laplacian phase unwrapping, projection onto dipole field (PDF) background field removal, and inversion solver to create the QSM image. Results consist of the MATLAB workspace, which contains the interim and final data, and a DICOM image. To create a DICOM image compatible with our PACS, we copy the header from the raw GRE data, change the title and series number, and add the QSM image.

#### 2.4.2. Key Considerations and Advice:

- Spending time up-front on design and deployment of robust and flexible infrastructure will ultimately save time and money in the long run.
- Suitably flexible infrastructure can be shared with other advanced sequence processing, e.g., arterial spin labeling, cerebrovascular reactivity, T2-relaxation under tagging, resting state functional MRI.
- Compartmentalizing infrastructure as virtual machines or containers helps manage the diversity of software requirements for various software.
- Have a plan for versioning your entire preprocessing processing pipeline and logging the code version(s) used for each reconstruction.

### 2.5. Image Postprocessing and Data Analysis

Our postprocessing scripts push the finalized QSM DICOM images back to our Research PACS. We use an extant DICOM viewer (Horos, Purview, Annapolis) to view our final data. Optionally, the images can then be pushed to the clinical PACS. These DICOM viewers offer many useful tools that facilitate inspection and analyses of the images, grading their quality, and otherwise interpreting them in clinical or research contexts. HOROS also allows us to save the spatial coordinates of regions of interest. Capturing the coordinates of these regions affords us the opportunity to place these regions of interest once and reusing them in the future when the expected improvements to QSM image processing come to fruition. These improvements may salvage poor (unusual) QSM data or improve already acceptable QSM images.

#### Key Considerations and Advice:

- Develop benchmarks for deciding when to adopt new image processing to salvage poor data or improve new data
- Develop code for two or more processing pipelines, and versioning (see below) for each.
- Retain the coordinates for regions of interest to evaluate new processing and to mitigate the amount of repeated work.
- Use full-featured DICOM viewers for reviewing the final images.

### 2.6. Code Versioning

Versioning code allows for updates to the processing pipeline and maintains a history of changes. We selected ‘git’ as our version control system because it is open source, familiar to most developers, integrates with many other tools, and works with either local or remote servers.

- Version both obvious tools (code) and non-obvious tools (e.g., acquisitions, databases, spreadsheets, standard operating procedures)
- Plan to periodically audit the use of the versioning tools, particularly for non-obvious tools.

## 3. Results

**Figure 3:**
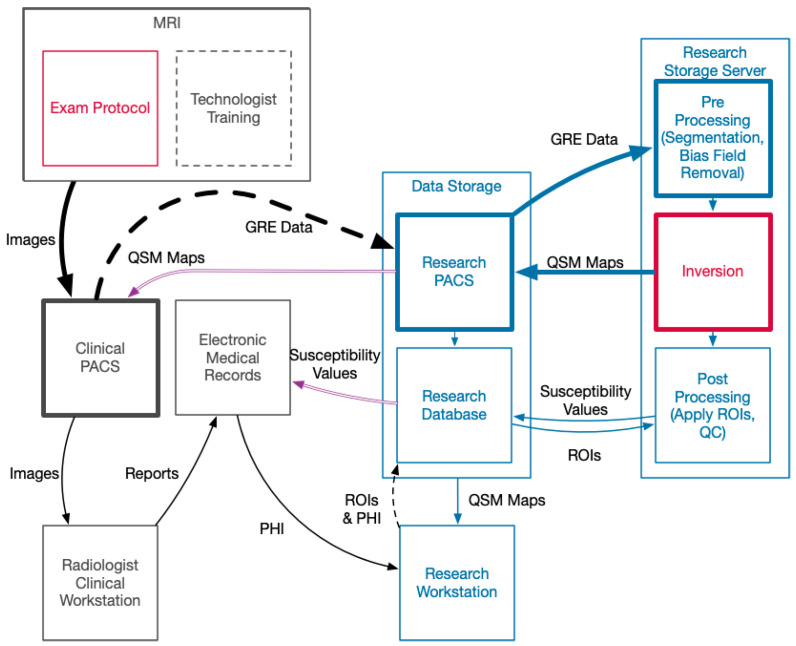
Diagram of data flow starting with acquisition. This process flow diagram shows a representative diagram of the key elements. The flow of data from acquisition to the generation of a QSM map is noted with bold lines. Red indicates aspects of QSM imaging covered in most imaging papers. Blue indicates research hardware, software, and data that facilitate the QSM imaging process. Purple indicates the clinically useful outputs of the research processing. Black are standard hospital systems and processes. Dashed lines indicate manual processes of training and data transfer wherein failures may cause data loss. Abbreviations: Magnetic Resonance Imaging (MRI), Gradient Recalled Echo (GRE), Picture Archive and Communication System (PACS), Quantitative Susceptibility Map (QSM), Quality Control (QC), Regions of Interest (ROI), Protected Health Information (PHI).

Developing a robust pipeline comes with implementation of a significant amount of hardware and software that is not standard in a clinical setting. The interaction between hospital-managed systems and our QSM infrastructure is summarized in Figure 3. A summary of the hardware and software implemented outside of the services provided by hospital IT is described in Table 2.

**Figure 4:**
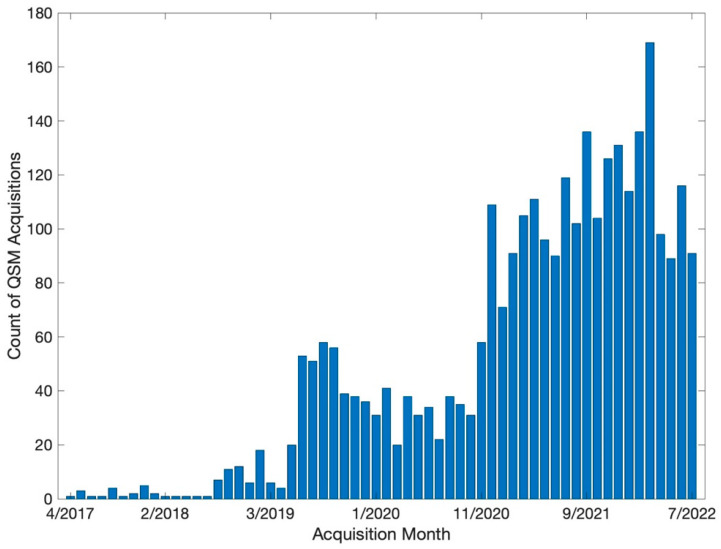
Number of cases acquired versus month they were acquired. The workflow and processes we describe are sufficient for high-volume acquisition and analysis of QSM data. This diagram shows a generally linear trend in study volume since 2019, with an interruption during the stages of the COVID-19 pandemic.

Our volume of QSM acquisitions of has grown quickly. Our initial acquisition was performed in April 2017, and we subsequently collected 3023 QSM series (Figure 4). Roughly 20% (N = 631) of these QSM exams were from patients of interest (See Figure 1 and Figure 2), for whom we processed their QSM data into QSM images. Our 24-core system can analyze approximately 100 datasets per day. Roughly 25% (N = 154) of the processed QSM exams were from patients for whom we also extracted medical histories for further analyses. Extracting medical histories takes approximately 1.5 h of manual effort per patient, with significant variation due to clinical complexity, origin of clinical notes, and the skill of the person conducting the data extraction process. Thus, our data acquisition and analysis pipeline meet the clinical demand in our Radiology Department, however our ability to extract medical histories into useable data is a rate limiting step for our research.

## 4. Discussion

An advanced imaging pipeline for QSM acquisition and processing can exist within a busy Radiology Department. Verifying the minimum quality of the image acquisition and integrity of the data storage are the two first steps. If the clinical PACS is the location to send the QSM data, then this simplifies the technologist workflow, minimizes the chance for data loss, and allows both the GRE magnitude and susceptibility images to be used in a clinical context. The focus of our report are the processing and analysis stools not covered by most reports on QSM; most of these tools can be assembled from extant tools routinely used in the engineering, medical, and technology sectors. Other tools will need to be created at each institution to cover their unique use-case. The acquisition or processing specific to QSM will likely be improved by other researchers and our workflow is designed to accept and implement their improvements. Our workflow is also extensible to other imaging that MR vendors do not support, including arterial spin labeling, T2-relaxation under tagging, resting state functional MRI, MR spectroscopy, and arbitrary non-cartesian and fast imaging techniques.

Optimized solutions can be achieved without much added cost as there are free and open-source options available that can be implemented wherever possible as this reduces the long-term cost and prevents vendor lock-in while maximizing compatibility and accessibility. Furthermore, developing in-house solutions can build the system faster and respond to challenges of getting data out of existing clinical and research servers. With image reconstruction time presenting a major consideration for clinical workflow integration, in-house solutions can be upgraded more rapidly to leverage speed improvements of new hardware or improved software. Using on-site storage can ease the institutional review board process as the data is physically protected. This can also lead to faster reconfiguration of systems for the inevitable challenges that arise when scaling a project from a single researcher’s computer to a distributed computing effort. 

Additionally, a modular approach can help decouple the software and hardware requirements and to reduce dependencies between components. It is possible to run all requisite components on a single physical host and operating system. However, this monolithic approach causes future issues such as diverging upgrade paths for various pieces of software and poor scalability while also creating a single point of failure. By using a virtualization platform such as Proxmox, VMWare, or Xen, and/or a containerization platform like Docker or Kubernetes, each software computing component can live as its own virtual machine or container. In such a design, all the components can still run on a single host if desired. However, segmenting resources provides significant flexibility regarding maintenance and scalability. As the project grows, increasing capacity is a matter of adding commodity hardware and migrating or replicating virtual machines rather than a complete reconfiguration of a single system. Further, as the project evolves or the system needs to take on new projects and roles, it is far simpler to add the required virtual machines without adding hardware.

### 4.1. Pitfalls

Storage design is the main pitfall for this project. These systems will be receiving information from scanners and PACS systems on a daily or on-going basis and reliability and redundancy are as important as their storage capacity. We overcome this pitfall by using the clinical PACS as our initial data destination. This leverages the hospital infrastructure to create a reliable first copy of our data. However, we also created high-reliability systems to service the remainder of the infrastructure. A second pitfall is the capture of non-imaging data. Complex medical histories need relational databases, and the labor-intensive data entry can only be scaled if multiple teams can access and manage data at the same time. We overcame this pitfall by deploying carefully constructed spreadsheets at the start of our project, with a database designed to subsume the data as the project scaled. The third pitfall is the versioning of all key components. Scaling and project longevity both require the ability to track and deploy changes over time. We overcame this by using extant development tools and agile project management for all aspects of this project. 

### 4.2. Limitations

Building and running such a system requires considerable knowledge and interfacing with the Department of Information Services. Depending on the scale of the project, a full-time engineer may be needed to complete the initial infrastructure setup and perform ongoing maintenance. Investigators lacking engineering experience can consider commercial options to replace some or all of these solutions. Some commercial systems are provided as a Software-as-a-Service model, which reduces the technical burden on research personnel and provides the safety net of a dedicated support team. They do present additional administrative challenges, such as obtaining institutional approval to host data off-site, as well as on-going maintenance costs. 

Our solution includes manual processes that require procedures and plans. Our data processing is suitable for the clinical needs, but our research use cases are bottlenecked by the efforts to place regions of interest and extract medical histories. The development of these processes requires familiarity with the neuroanatomy, DICOM viewers, and electronic medical records. While investigators typically know these quite well, as the project scales the work can fall to others who may introduce various errors. A rigorously defined manual for training and operating these processes are essential tools.

## 5. Conclusions

QSM is an imaging modality ready to cross from research tool to clinical practice. The lack of vendor support for its acquisition and analysis requires deploying a custom solution to process these data. This manuscript serves to highlight the clinical utility of QSM and provide a guide for clinicians and investigators interested in using QSM as part of routine clinical imaging. Our approach is already creating clinical data that impacts the diagnosis and management of patients with iron related pathologies. Many similar imaging modalities exist, this manuscript and the tools it outlines are suitable for adaptation and processing many other types of medical images.

## Figures and Tables

**Table 1:** Scan parameters.

Parameter	MEDI Toolbox Recommendations	CHLA QSM Protocol
**TE**	4 echoes, minimum first TE with uniform spacing	4 echoes, first TE = 5.7 ms, 6.9 ms echo spacing
**TR**	Minimum (50–60 ms)	(Shortest) 31 ms
**Flip angle**	20	17
**BW**	150 Hz/px	189.6 Hz/px
**FOV**	240 mm × 240 mm	210 mm (AP) × 189 mm (RL)
**Number of Slices**	50–60 (or as many to achieve whole brain coverage if needed)	84
**Slice Thickness**	2 mm or thinner if timing allows	1.3 mm
**Frequency**	>400	212
**Phase**	>300	189
**NSA**	0.75	1
**SENSE**	Allowed	Yes, factor = 2 (P), 1.29 (S)

**Table 2:** Scan parameters.

Physical Hardware	Specification	Use
Apple iMac	MacOS Intel i7 16 GB RAM	Image segmentation via Horos
2-blade enterprise server	Dual Intel Xeon Gold 6148F processors 40 cores/80 threads per blade 256 GB RAM/nod	Host virtual machines Provide distributed file system
Synology NAS	14 TB available storage	Provide storage redundancy
Network Switch	10 Gigabit (10GBASE-T)	Inter-network communication Support distributed file system
**Virtual Machine**	**OS and Software**	**Use**
Research PACS Server	Ubuntu 20.04 LTS Orthanc DICOM server DCMTK (DICOM toolkit for interacting with certain PACS servers) Bash (scripting) Python3 (scripting) Mysql client Git (code versioning)	Image storage Download from clinical PACS Serving images to processing systems
Research Database Server	Debian 11 MariaDB (mysql) server Phpmyadmin (manual data entry, database maintenance)	Storage of clinical review information Storage of PACs and image processing databases
Processing Server	Ubuntu 20.04 LTS with KDE Plasma Desktop 24 virtual processor cores, 128GB RAM, 128GB disk Ubuntu 20.04 LTS with Desktop environment MATLAB 2021b Python3 Bash Git	Execute processing code Store results on successful completion
MATLAB License Server	Ubuntu 20.04LTS MATLAB FlexLM	Provide MATLAB licenses to processing systems

## Supplementary Materials

Example code describing database setup and image processing can be found at https://github.com/cornercase/qsm-manuscript-example-scripts.

## References

[1] Harada T., Kudo K., Fujima N., Yoshikawa M., Ikebe Y., Sato R., Shirai T., Bito Y., Uwano I., Miyata M. (2022). Quantitative Susceptibility Mapping: Basic Methods and Clinical Applications. RadioGraphics.

[2] Hobson N., Polster S.P., Cao Y., Flemming K., Shu Y., Huston J., Gerrard C.Y., Selwyn R., Mabray M., Zafar A. (2020). Phantom validation of quantitative susceptibility and dynamic contrast-enhanced permeability MR sequences across instruments and sites. J. Magn. Reson. Imaging.

[3] Deh K., Zaman M., Vedvyas Y., Liu Z., Gillen K.M., O’ Malley P., Bedretdinova D., Nguyen T., Lee R., Spincemaille P. (2020). Validation of MRI quantitative susceptibility mapping of superparamagnetic iron oxide nanoparticles for hyperthermia applications in live subjects. Sci. Rep..

[4] Wang C., Martins-Bach A.B., Alfaro-Almagro F., Douaud G., Klein J.C., Llera A., Fiscone C., Bowtell R., Elliott L.T., Smith S.M. (2022). Phenotypic and genetic associations of quantitative magnetic susceptibility in UK Biobank brain imaging. Nat. Neurosci..

[5] Langkammer C., Schweser F., Krebs N., Deistung A., Goessler W., Scheurer E., Sommer K., Reishofer G., Yen K., Fazekas F. (2012). Quantitative susceptibility mapping (QSM) as a means to measure brain iron? A post mortem validation study. NeuroImage.

[6] Wu B., Li W., Avram A.V., Gho S.-M., Liu C. (2012). Fast and tissue-optimized mapping of magnetic susceptibility and T2* with multi-echo and multi-shot spirals. NeuroImage.

[7] Bandt S.K., de Rochefort L., Chen W., Dimov A.V., Spincemaille P., Kopell B.H., Gupta A., Wang Y. (2019). Clinical Integration of Quantitative Susceptibility Mapping Magnetic Resonance Imaging into Neurosurgical Practice. World Neurosurg..

[8] de Rochefort L., Brown R., Prince M.R., Wang Y. (2008). Quantitative MR susceptibility mapping using piece-wise constant regularized inversion of the magnetic field. Magn. Reson. Med..

[9] Shmueli K., de Zwart J.A., van Gelderen P., Li T.-Q., Dodd S.J., Duyn J.H. (2009). Magnetic susceptibility mapping of brain tissue in vivo using MRI phase data. Magn. Reson. Med..

[10] Carey M.P., Burish T.G. (1988). Etiology and treatment of the psychological side effects associated with cancer chemotherapy: A critical review and discussion. Psychol. Bull..

[11] Liu J., Liu T., de Rochefort L., Ledoux J., Khalidov I., Chen W., Tsiouris A.J., Wisnieff C., Spincemaille P., Prince M.R. (2012). Morphology Enabled Dipole Inversion for Quantitative Susceptibility Mapping Using Structural Consistency Between the Magnitude Image and the Susceptibility Map. Neuroimage.

[12] Marques J.P., Bowtell R. (2005). Application of a Fourier-based method for rapid calculation of field inhomogeneity due to spatial variation of magnetic susceptibility. Concepts Magn. Reson. Part B Magn. Reson. Eng..

[13] Salomir R., de Senneville B.D., Moonen C.T. (2003). A fast calculation method for magnetic field inhomogeneity due to an arbitrary distribution of bulk susceptibility. Concepts Magn. Reson. Part B Magn. Reson. Eng..

[14] Ferreira A., Neves P., Gozzelino R. (2019). Multilevel Impacts of Iron in the Brain: The Cross Talk between Neurophysiological Mechanisms, Cognition, and Social Behavior. Pharm. Basel Switz..

[15] Jáuregui-Lobera I. (2014). Iron deficiency and cognitive functions. Neuropsychiatr. Dis. Treat..

[16] Tang S., Nie L., Liu X., Chen Z., Zhou Y., Pan Z., He L. (2022). Application of Quantitative Magnetic Resonance Imaging in the Diagnosis of Autism in Children. Front. Med..

[17] Thomas G.E.C., Zarkali A., Ryten M., Shmueli K., Gil-Martinez A.L., Leyland L.-A., McColgan P., Acosta-Cabronero J., Lees A.J., Weil R.S. (2021). Regional brain iron and gene expression provide insights into neurodegeneration in Parkinson’s disease. Brain J. Neurol..

[18] Wang J.-Y., Zhuang Q.-Q., Zhu L.-B., Zhu H., Li T., Li R., Chen S.-F., Huang C.-P., Zhang X., Zhu J.-H. (2016). Meta-analysis of brain iron levels of Parkinson’s disease patients determined by postmortem and MRI measurements. Sci. Rep..

[19] Acosta-Cabronero J., Machts J., Schreiber S., Abdulla S., Kollewe K., Petri S., Spotorno N., Kaufmann J., Heinze H.-J., Dengler R. (2018). Quantitative Susceptibility MRI to Detect Brain Iron in Amyotrophic Lateral Sclerosis. Radiology.

[20] Dusek P., Hofer T., Alexander J., Roos P.M., Aaseth J.O. (2022). Cerebral Iron Deposition in Neurodegeneration. Biomolecules.

[21] Miao X., Choi S., Tamrazi B., Chai Y., Vu C., Coates T.D., Wood J.C. (2018). Increased brain iron deposition in patients with sickle cell disease: An MRI quantitative susceptibility mapping study. Blood.

[22] Ravanfar P., Loi S.M., Syeda W.T., Van Rheenen T.E., Bush A.I., Desmond P., Cropley V.L., Lane D.J.R., Opazo C.M., Moffat B.A. (2021). Systematic Review: Quantitative Susceptibility Mapping (QSM) of Brain Iron Profile in Neurodegenerative Diseases. Front. Neurosci..

[23] Cossu G., Abbruzzese G., Matta G., Murgia D., Melis M., Ricchi V., Galanello R., Barella S., Origa R., Balocco M. (2014). Efficacy and safety of deferiprone for the treatment of pantothenate kinase-associated neurodegeneration (PKAN) and neurodegeneration with brain iron accumulation (NBIA): Results from a four years follow-up. Parkinsonism Relat. Disord..

[24] Ivankovic M., Radman M., Gverovic-Antunica A., Tesanovic S., Trgo G., Demarin V. (2013). Influence of hypertension and type 2 diabetes mellitus on cerebrovascular reactivity in diabetics with retinopathy. Ann. Saudi Med..

[25] Hider R.C., Hoffbrand A.V. (2018). The Role of Deferiprone in Iron Chelation. N. Engl. J. Med..

[26] Valdés Hernández M.d.C., Case T., Chappell F.M., Glatz A., Makin S., Doubal F., Wardlaw J.M. (2019). Association between Striatal Brain Iron Deposition, Microbleeds and Cognition 1 Year After a Minor Ischaemic Stroke. Int. J. Mol. Sci..

[27] Martinez-Ramirez S., Greenberg S.M., Viswanathan A. (2014). Cerebral microbleeds: Overview and implications in cognitive impairment. Alzheimers Res. Ther..

[28] Kłos J., van Laar P.J., Sinnige P.F., Enting R.H., Kramer M.C.A., van der Weide H.L., van Buchem M.A., Dierckx R.A.J.O., Borra R.J.H., van der Hoorn A. (2019). Quantifying effects of radiotherapy-induced microvascular injury; review of established and emerging brain MRI techniques. Radiother. Oncol. J. Eur. Soc. Ther. Radiol. Oncol..

[29] ADNI | ADNI 3. https://adni.loni.usc.edu/adni-3/.

[30] MarkVCID1 Protocols & Resources | MarkVCID. https://markvcid.partners.org/markvcid1-protocols-resources.

